# Association between FSHR polymorphisms and polycystic ovary syndrome among Chinese women in north China

**DOI:** 10.1007/s10815-013-0166-z

**Published:** 2014-01-05

**Authors:** Xue-qing Wu, Su-ming Xu, Jun-fen Liu, Xing-yu Bi, Yuan-xia Wu, Jing Liu

**Affiliations:** Center of Reproductive Medicine, Shanxi Women and Children Health Hospital, No. 13, Xin Min Bei Jie, Xinghualing District, 030013 Taiyuan, Shanxi China

**Keywords:** Polycystic ovary syndrome, Follicle-stimulating hormone receptor, Genetic polymorphisms, Single nucleotide polymorphisms

## Abstract

**Purpose:**

Polycystic ovary syndrome (PCOS) is a common endocrine disorder disease among women in reproductive-age. Since follicle stimulating hormone (FSH) exerts important biological functions, the association between PCOS and FSH receptor (FSHR) polymorphisms attracts wide attention. The aim of this study was to evaluate whether polymorphisms of FSHR at 307 and 680 codons are associated with PCOS patients in China.

**Methods:**

Patients with PCOS (*n* = 215) and controls (*n* = 205) were recruited from Shanxi Province in north China. They are Han ethnics. Genomic DNA was isolated from the venous blood. The Ala307Thr and Ser680Asn polymorphisms of FSHR were analyzed by polymerase chain reaction–restriction fragment length polymorphism (PCR–RFLP) and direct DNA sequencing.

**Results:**

The distributions of genotype and allele of Ala307Thr and Ser680Asn polymorphisms of FSHR were not statistically different between the PCOS patients and the controls. Analysis of the frequency of FSHR polymorphisms showed no statistical difference among the PCOS patients with different obesity standards. Although there were no statistical differences in the most of the endocrine parameters including LH, LH/FSH, E2, P and T as well as the clinical pregnancy rate, there were significant differences in the levels of FSH and PRL among PCOS patients carrying different genotypes of Ala307Thr and Ser680Asn polymorphisms.

**Conclusion:**

The Ala307Thr and Ser680Asn polymorphisms of FSHR are not associated with PCOS in Han ethnic Chinese women in north China. The FSHR polymorphisms was related to the levels of FSH and PRL but not other PCOS-associated endocrine hormones as well as clinical pregnancy rate in PCOS patients of Han Chinese ethnical population.

## Introduction

Polycystic ovary syndrome (PCOS) is a common endocrine disorder disease among women of reproductive age. The prevalence of PCOS is 5–10 % and the clinical manifestation of the disease is diverse [1]. According to the 2003 Rotterdam criteria [2], the characteristics of PCOS mainly include oligomenorrhea or amenorrhea, hyperandrogenism and polycystic ovary morphology. The pathogenesis of PCOS is not fully understood. However, the influence of heredity and environment is considered to be the potential causative factors for the disease. Recent studies revealed that there are multiple susceptibility genes associated with PCOS, including growth hormone receptor exon 3 [3], CYP11A1 [4] and GnRH [5].

Follicle stimulating hormone (FSH) is an important hormone for women and plays a role in the follicle development, oocyte maturation, and steroidogenesis regulation [6]. It mediates FSH receptor (FSHR) through its biological functions [7]. FSHR belongs to the G-protein coupled receptor family and consists of 10 exons, 9 introns and the promoter region at chromosome 2p21 [8]. The level of FSH is controlled by FSHR, and aberrant FSHR affects ovary and folliculogenesis [6]. Previous studies revealed a low incidence of gene mutations for the FSHR gene in nature [9]. Single nucleotide polymorphisms (SNP) were identified in the FSHR gene [10, 11]. Among them, Ala307Thr and Ser680Asn polymorphisms in exon 10 have drawn increasing attention. Due to the changes of nucleotide at 307 and 680 loci, which lead to changes of corresponding amino acids, FSHR varies in its biological effects. Many studies showed that FSHR polymorphisms at positions 307 and 680 may be even more relevant for clinical practice for the reasons of regulating the ovarian reaction to hormone, controlling ovarian hyperstimulation, changing the menstrual cycle, and causing premature ovarian failure (POF) and PCOS [12]. However, some researches suggested that the results were inconsistent with the interrelationship between FSHR polymorphisms and PCOS. Gut et al. reported that the Ser680Asn of FSHR was associated with PCOS in Korean women, whereas the Ala307Thr was not [6]. On the contrary, a research, done by Dolfin et al., showed that the Ala307Thr of FSHR polymorphism was related to PCOS in Italian women [13]. In addition, Unsal et al. found that the genotye frequencies of the Ala307Thr and Ser680Asn polymorphisms of FSHR were not different between the cases and controls in Turkish adolescent girls [14]. In a recent study, we also found that the association between FSHR polymorphisms and PCOS in China was also lack of consistency [15, 16]. Since these studies were conducted in different geographic regions and with different ethnic groups, the discrepant results suggested the FSHR–PCOS interrelationship to be geography and race specific. In this study, we investigated Ala307Thr and Ser680Asn polymorphisms of the FSHR gene and the relationship between FSHR polymorphisms and PCOS in the Han Chinese women in north China.

## Materials and methods

### Subjects

Both the PCOS patients (*n* = 215) and the controls (*n* = 205) were recruited by Reproductive Medicine Center, Shanxi Women and Infants Hospital, Taiyuan, China between January 2012 and January 2013. All subjects were Han ethnic, from the Shaanxi Province, in north China. The study was approved by the local Medical Ethical Committee and the people of the PCOS and the control was consented.

The patients were selected based on the 2003 Rotterdam criteria [2]. Meanwhile, other diseases which could cause hyperandrogenism, such as congenital adrenal hyperplasia, hypothyroidism, androgen-secreting tumors, Cushing’s syndrome, were excluded.

The controls were infertility patients due to tubal and or male factors with normal ovarian function.

All of the patients had been ruled out of thyroid abnormalities, cardiovascular system diseases or other endocrine metabolic disorder disease.

### Clinical measurements

The general medical information was collected and recorded, including the clinical examination results: age, height, weight, waist circumference, hip circumference, menstrual history of fertility, B ultrasound assessment, etc. And the body mass index (BMI) and waist/hip ratio (WHR) were calculated accordingly. The clinical hyperandrogenism was assessed by the modified Ferriman–Gallwey score (mF–G score) [17]. Transvaginal ultrasonography was used to identify polycystic ovaries (PCO) on a Aloka Prosound α10 (Tokyo, Japan) ultrasound machine. According to the description of Xu [18], all the above clinical measurements were completed by two physicians. The mean value of what was calculated.

### Biochemical measurements

On the 3rd to the 5th day of menstrual cycles, blood samples were taken between 8 AM and 9 AM after a 12-h overnight fast and immediately centrifuged and frozen at −80 °C until assayed. For the amenorrhea patients, blood samples were taken on any day of menstruation providing no dominat follicle detected under B-ultrasound. The hormone levels, including follicle-stimulating hormone (FSH, Reference Value: 4.5 ~ 11.0 mIU/ml), luteinizing hormone (LH, Reference Value:1.7 ~ 13.3 mIU/ml), estradiol (E2, Reference Value: 40.7 ~ 220.4 pg/ml), progesterone (P, Reference Value: ≤0.87 ng/ml), prolactin (PRL, Reference Value: 4.1 ~ 28.9 ng/ml), and testosterone (T, Reference Value: 9.8 ~ 82.1 ng/dl) were measured by AIA-200 ST Automated Immunoassay Analyzer (Tosoh Corporation, Tokyo, Japan). The cut-off level of testosterone >82.1 ng/dL was defined as biochemical hyperandrogenism.

Plasma glucose (Reference Value: 3.90 ~ 6.10 mmol/L) was detected by the hexokinase (HK) method, and insulin (Reference Value: 0 ~ 28.4 uIU/ml) was detected by the chemiluminescence method. The homeostatic model assessment for insulin resistance (HOMA-IR) was calculated as follows: (fasting insulin × fasting glucose)/22.5. Serum cholesterol (CHO, Reference Value: ≤5.2 mmol/L), triglycerides (TG, Reference Value: ≤1.7 mmol/L), high-density lipoprotein-cholesterol (HDL, Reference Value: >1.03 mmol/L) and low-density lipoprotein (LDL, Reference Value: <3.34 mmol/L) levels were measured using the precipitation and enzymatic method.

### Genotyping

Genomic DNA was extracted from heparinized venous blood using E.Z.N.A Blood DNA Kit (Omega, Bio-Tek, Norcross, GA, USA) according to the manufacturer’s instructions. Polymerase chain reaction (PCR) was performed in a total volume of 50 μl containing 100 ng of genomic DNA, 0.3 M of forward and reverse primers, 0.2 mM of dNTP, 2.5 unit of Taq DNA polymerase, and 2 mM of MgSO_4_. The PCR amplification was initiated at 94 °C for 5 min, followed by 30 cycles consisting of denaturing at 94 °C for 30 s, annealing at 55 °C (for Ala307Thr) or 60 °C (for Ser680Asn) for 30 s, and elongation at 72 °C for 1 min, and a final step at 72 °C for 10 min. All PCR products were subjected to direct DNA sequencing, and 15 % of PCR products were subjected to restriction enzyme digestion for validation by PCR-restriction fragment length polymorphism (PCR-RFLP). Restriction enzymes Ahd I and Bsr I (all from New England Biolabs, Ipswich, MA, USA) were used for Ala307Thr and Ser680Asn, respectively. Ahd I digestion was performed at 37 °C for 4 h, followed by inactivation at 65 °C for 20 min. Bsr I digestion was performed at 65 °C for 4 h, followed by inactivation at 80 °C for 20 min. Both PCR and enzyme digested products were evaluated by electrophoresis in 2 % and 4 % agarose gels, respectively. The PCR primers, annealing temperatures, product sizes, restriction enzymes, and allele sizes are shown in Table 1 [19, 20].

### Statistical analysis

Statistical analysis was performed using SPSS Statistics Version 13.0. Student’s *t*-test or Mann–Whitney *U* test was used to analyze the differences of two continuous variables. ANOVA and Kruskal–Wallis tests were used to analyze the differences among multiple (>2) groups. Genotype frequencies were tested for Hardy–Weinberg equilibrium. Chi-squared test was used to analyze the differences in genotype and allele frequencies between PCOS and controls. Results were expressed as percentage and/or mean ± standard deviation (SD). A *p*-value <0.05 was considered statistically significant. Linkage disequilibrium (LD) was estimated using D’ and r^2^ on SHEsis (http://analysis2.bio-x.cn/myAnalysis.php).

## Results

A total of 215 PCOS patients and 205 controls were recruited in this study. All subjects were Han ethnics in Shanxi Province of north China. The baseline characteristics of the subjects were shown in Table 2. The levels of Weight, BMI, WHR, LH, LH/FSH, T, Fasting glucose, HOMA-IR and TG were higher whereas the levels of Menarche age, FSH, PRL, HDL and LDL were lower in the PCOS patients than in the controls. There were no significant differences between the PCOS patients and the controls in Age, Height, Waist, Hip, mF-G score, E2, P, Fasting insulin and CHO (Table 1).

**Table 1:** Primers, annealing temperatures, product sizes, restriction enzymes, and allele sizes

Polymorphism	Primer sequence (5′-3′)	Tm (°C)	Product size	Restriction enzyme	Allele size	Reference
Ala307Thr	F: 5′-CCTGCACAAAGACAGTGATG-3′;	55	577	AhdI	Ala:403 + 174	[19]
	R: 5′-TGGCAAAGACAGTGAAAAG-3′				Thr:403 + 143 + 31	
Ser680Asn	F: 5′-TTTGTGGTCATCTGTGGCTGC-3′;	60	520	BsrI	Asn:520	[20]
	R:5′-CAAAGGCAAGGACTGAATTATCATT-3′				Ser:413 + 107	

*F* forward primer, *R* reverse primer, *Tm* annealing temperatures, *R* reference

**Table 2:** Baseline characteristics of all subjects

Variable	Controls (*n* = 205)	Cases (*n* = 215)	*p* value
Age (year)	31.06 ± 4.89	30.02 ± 4.92	0.163^a^
Weight (kg)	59.19 ± 7.75	63.40 ± 11.31	0.023^b^
Height (cm)	161.60 ± 4.45	161.66 ± 5.70	0.979^b^
BMI (kg/m^2^)	22.77 ± 3.96	24.49 ± 4.26	0.007^a^
Waist (cm)	80.50 ± 7.88	92.38 ± 9.46	0.058^a^
Hip (cm)	96.75 ± 7.52	103.59 ± 6.92	0.076^a^
WHR	0.83 ± 0.06	0.89 ± 0.07	0.009^a^
Menarche age (year)	13.66 ± 1.26	13.04 ± 1.20	0.001^b^
mF-G score	8.51 ± 0.09	8.79 ± 1.20	0.678^b^
FSH (mIU/ml)	8.81 ± 5.96	8.79 ± 10.09	0.009^b^
LH (mIU/ml)	5.44 ± 3.97	7.23 ± 4.36	<0.001^b^
LH/FSH	0.81 ± 0.76	1.04 ± 4.36	<0.001^b^
E2 (pg/ml)	56.99 ± 34.51	55.45 ± 24.82	0.385^b^
P (ng/ml)	0.85 ± 2.62	2.39 ± 10.82	0.441^b^
T (ng/dl)	37.52 ± 19.31	54.17 ± 27.70	<0.001^b^
PRL (ng/ml)	16.74 ± 9.72	14.84 ± 12.32	0.026^b^
Fasting insulin (mIU/ml)	6.92 ± 3.57	10.97 ± 6.45	0.727^b^
Fasting glucose (mmol/l)	5.13 ± 0.58	5.53 ± 0.94	0.015^b^
HOMA-IR	1.45 ± 1.13	2.17 ± 1.65	<0.001^b^
CHO (mmol/l)	4.42 ± 0.64	4.17 ± 0.36	0.058^a^
TG (mmol/l)	1.34 ± 0.16	1.88 ± 1.20	0.027^b^
HDL (mmol/l)	1.43 ± 0.59	1.20 ± 0.23	0.022^a^
LDL (mmol/l)	2.44 ± 0.44	2.25 ± 0.15	0.007^a^

*BMI* body mass index, *WHR* waist/hip ratio, *mF–G score* modified Ferriman–Gallwey score, *FSH* follicle-stimulating hormone, *LH* luteinizing hormone, *E2* estradiol, *P* progesterone, *T* testosterone, *PRL* prolactin, *HOMA-IR* homoeostasis model assessment, *CHO* cholesterol, *TG* triglycerides, *HDL* high-density lipoprotein, *LDL* low-density lipoprotein

^a^Student’s *t*-test

^b^Mann–Whtitney *U* test

**Fig. 1:**
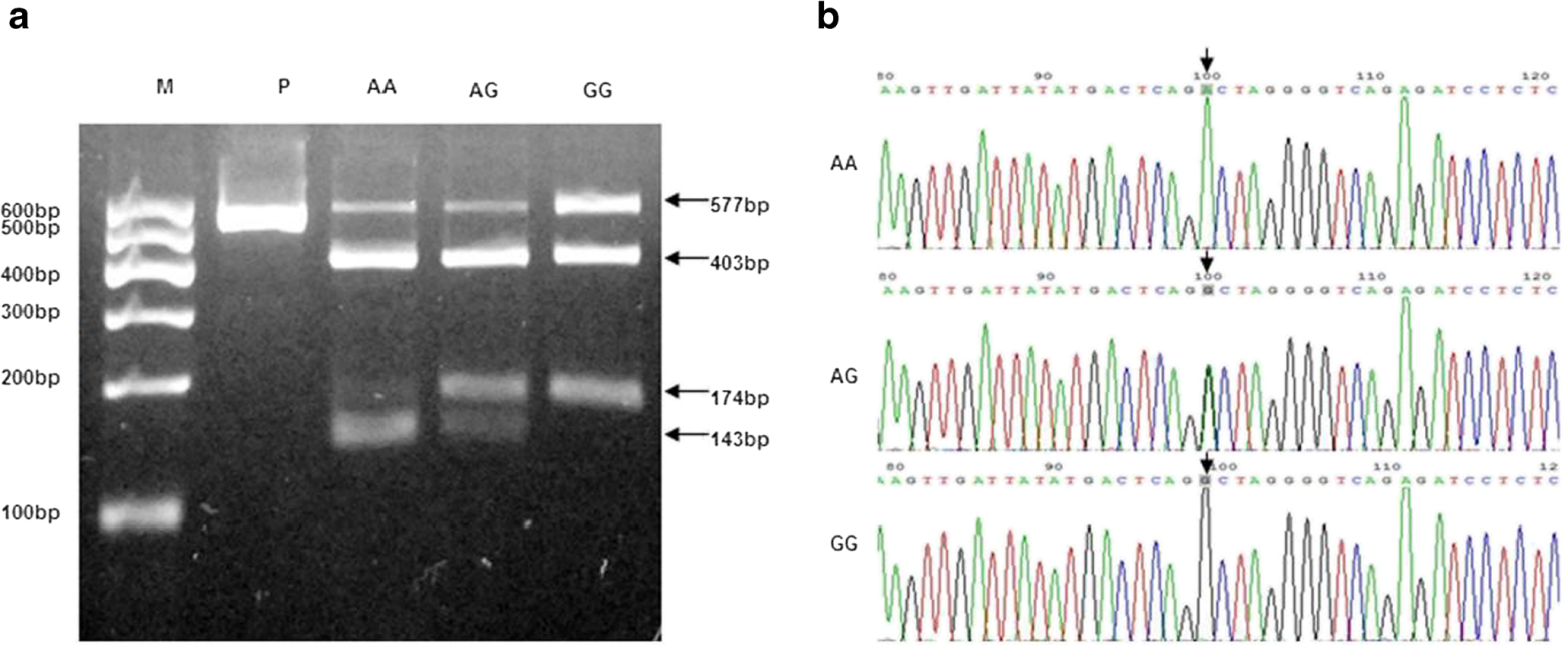
**a** Electrophoretogram of DNA fragments for Ala307Thr polymorphisms after digestion with Ahd I. Homozygote A/A was shown by the bands of 403 bp, 143 bp and 31 bp. Homozygote G/G was shown by the bands of 403 bp and 174 bp. Heterozygote A/G was shown by the bands of 403 bp, 174 bp, 143 bp and 31 bp. Note that the 31 bp bands were run off the gel. **b** DNA sequencing of the Ala307Thr polymorphisms (AA, AG and GG) as indicated by *arrows*. The lane marked M is the marker of DNA. The lane marked P is the DNA of PCOS without enzyme digestion

**Fig. 2:**
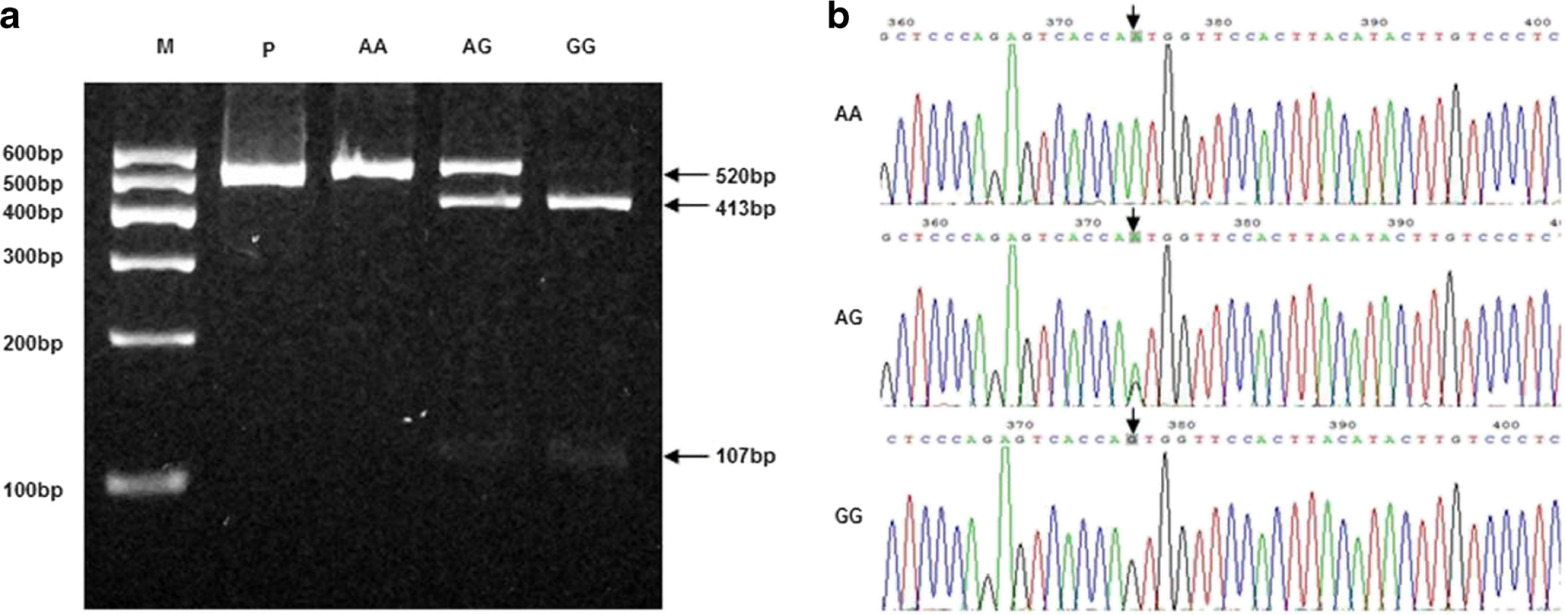
**a** Electrophoretogram of DNA fragments for Ser680Asn polymorphism after digestion with Bsr I. Homozygote A/A was shown by the band of 520 bp. Homozygote G/G was shown by the bands of 413 bp and 107 bp. Heterozygote A/G was shown by the bands of 520 bp, 413 bp and 107 bp. **b** DNA sequencing of the Ser680Asn polymorphisms (AA, AG and GG) as indicated by *arrows*. The lane marked M is the marker of DNA. The lane marked P is the DNA of PCOS without enzyme digestion

Ala307Thr and Ser680Asn polymorphisms of FSHR were analyzed by PCR–RFLP and DNA sequencing (Figs. 1 and 2). The distribution of FHSR genotypes and allele frequencies in PCOS patients and controls were evaluated (Table 3). The distribution of the polymorphisms was conformed by Hardy–Weinberg equilibrium test. In PCOS patients, linkage disequilibrium (D’) between the Ala307Thr and Asn680Ser of FSHR polymorphisms was 0.932 (*r* ^2^ = 0.831), indicating a near-complete linkage disequilibrium. However, there were no statistical differences in the allele or genotype frequencies of the polymorphisms between the PCOS patients and the controls.

**Table 3:** Genotype distribution and allele frequency of FSHR polymorphism

Genotype	Cases (*n* = 215)	Controls (*n* = 205)	*p* value
A307T(rs6165)			
Thr/Thr(AA)	93 (43.3 %)	91 (44.4 %)	0.133
Ala/Thr(AG)	95 (44.2 %)	100 (48.8 %)	
Ala/Ala(GG)	27 (12.6 %)	14 (6.8 %)	
H-W test	0.721	0.052	
Allele frequency			
Ala(G)	149 (15.3 %)	128 (31.5 %)	0.29
Thr(A)	281 (65.2 %)	282 (69.3 %)	
N680S(rs6166)			
Asn/Asn(AA)	93 (43.3 %)	94 (45.9 %)	0.069
Ser/Asn(AG)	94 (43.7 %)	98 (47.8 %)	
Ser/Ser(GG)	28 (13.0 %)	13 (6.3 %)	
H-W test	0.581	0.057	
Allele frequency			
Asn(A)	280 (69.8 %)	286 (69.8 %)	0.152
Ser(G)	150 (30.2 %)	124 (30.2 %)	

*p* values from chi-squared test

*H*–*W test* Hardy–Weinberg equilibrium test

**Fig. 3:**
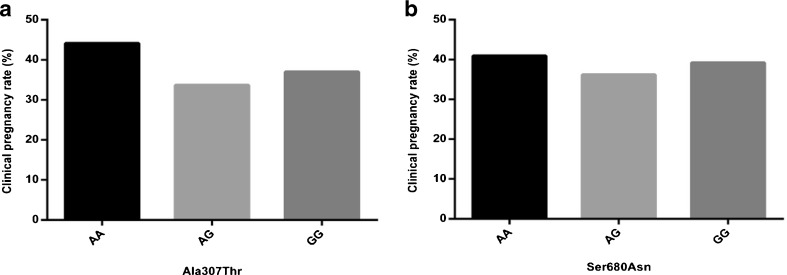
Clinical pregnancy rate of PCOS patients carrying different FSHR polymorphisms

We further analyzed the phenotypes for the Ala307Thr and Ser680Asn polymorphisms in the PCOS patients. There were no statistical differences among the different obesity standards of the PCOS patients with different FSHR polymorphisms (Table 4). For the endocrine parameters, there were significant differences in the level of FSH among the PCOS patients carrying different genotypes of the two polymorphisms (*p* = 0.027 for Ser680Asn; *p* = 0.009 for Ala307Thr) and in the level of PRL among the PCOS patients carrying different genotypes of the Ser680Asn polymorphisms (*p* = 0.009) (Table 5). There were no statistical differences in LH, LH/FSH, E2, P and T. Moreover, no statistical difference was found in the clinical pregnancy rate among the PCOS patients carrying different genotypes of the two polymorphisms (Fig. 3).

**Table 4:** FSHR polymorphisms versus different obesity standards in PCOS patients

	Ala307Thr				Ser680Asn			
	AA (*n* = 93)	AG (*n* = 95)	GG (*n* = 27)	*p* value	AA (*n* = 93)	AG (*n* = 94)	GG (*n* = 28)	*p* value
BMI								
<18.5	9 (52.9 %)	6 (35.3 %)	2 (11.8 %)	0.68	6 (50.0 %)	3 (25.0 %)	3 (25.0 %)	0.66
18.5–24	29 (36.2 %)	42 (52.5 %)	9 (11.2 %)		33 (41.8 %)	35 (44.3 %)	11 (13.9 %)	
24–28	35 (45.5 %)	31 (40.3 %)	11 (14.3 %)		29 (40.8 %)	32 (45.1 %)	10 (14.1 %)	
>28	20 (48.8 %)	16 (39.0 %)	5 (12.2 %)		25 (47.2 %)	24 (45.3 %)	4 (7.5 %)	
WHR								
<0.85	31 (48.4 %)	29 (45.3 %)	4 (6.2 %)	0.18	35 (47.9 %)	34 (46.6 %)	4 (5.5 %)	0.06
>0.85	62 (41.4 %)	66 (43.7 %)	23 (15.2 %)		58 (40.8 %)	60 (42.3 %)	24 (16.9 %)	

*p* values from chi-squared test on the different genotypes of FSHR polymorphisms

**Table 5:** FSHR polymorphisms versus endocrine parameters in PCOS patients

	Ala307Thr				Ser680Asn			
	AA (*n* = 93)	AG (*n* = 95)	GG (*n* = 27)	*p* value	AA (*n* = 93)	AG (*n* = 94)	GG (*n* = 28)	*p* value
FSH (mIU/ml)	7.07 ± 2.54	7.48 ± 1.77	13.22 ± 16.64	0.009	7.12 ± 2.55	7.57 ± 1.89	13.71 ± 17.46	0.027
LH (mIU/ml)	7.19 ± 4.48	7.88 ± 5.78	7.53 ± 4.71	0.823	7.14 ± 4.45	7.22 ± 4.69	8.45 ± 4.89	0.445
LH/FSH	1.11 ± 0.81	1.09 ± 0.79	0.89 ± 0.60	0.539	1.06 ± 0.66	0.99 ± 0.65	0.99 ± 0.63	0.924
E2 (pg/ml)	55.60 ± 15.43	55.32 ± 34.72	53.77 ± 19.81	0.301	55.59 ± 15.57	56.32 ± 32.99	52.55 ± 20.21	0.415
P (ng/ml)	1.30 ± 5.52	2.61 ± 11.32	4.65 ± 14.05	0.718	1.31 ± 5.64	0.79 ± 2.00	0.44 ± 0.13	0.927
T (ng/dl)	60.19 ± 29.37	51.91 ± 28.34	44.76 ± 24.86	0.115	59.40 ± 29.85	53.69 ± 27.62	45.52 ± 25.05	0.282
PRL (ng/ml)	17.20 ± 15.68	12.07 ± 7.38	16.55 ± 6.46	0.052	17.73 ± 15.69	11.70 ± 6.93	17.01 ± 6.80	0.009

*p* values from ANOVA test on the different genotypes of FSHR polymorphisms

## Discussion

It has been known that PCOS is a hereditary endocrine disease. With changes of living environment and increase of social pressure, the incidence of PCOS gradually increases during recent years. The clinical manifestations of PCOS patients appear diverse in different geographic regions and among different races. Up to date, knowledge about PCOS has been mainly obtained from the studies involving Western populations. A recent study comparing 547 Chinese women with 427 Dutch women revealed some differences in the phenotypic characteristics of PCOS between the two ethnic groups [21]. In this study, we assessed the baseline characteristics between the PCOS patients (*n* = 215) and the controls (*n* = 205) in the Han ethnic of Shanxi Province in north China. We observed increased levels of Weight, BMI, WHR, LH, LH/FSH, T, Fasting glucose, HOMA-IR and TG and decreased levels of Menarche age, FSH, PRL, HDL and LDL in the PCOS patients.

As a complex hereditary endocrine disease, analyses of major genes associated with PCOS have been attempted for better understanding of the etiology of the disease. Since it plays an important role in FSH signal transduction, FSHR has been extensively studied including its gene structure. The polymorphisms at the 307 and 680 loci on exon 10 of FSHR have become an attracting topic in recent years. A study from Shanghai, a metropolitan city in southeast China, showed a significant association between PCOS patients and Ser680Asn but not Ala307Thr polymorphisms of FSHR in the Han ethnic women [15]. To investigate whether this observation also exists in different geographic regions of China, we recruited patients with the same Han ethnic from Shanxi Province in north China. However, we failed to observe any association between PCOS patients and the Ser680Asn polymorphisms of FSHR. Our results are consistent with a recently reported study conducted in Xi’an, a city near Shanxi Province within the same north China region [16] and a study performed in a different geographic region (Netherlands) and with different ethnical women (Caucasian) [5]. Therefore, studies using different ethnical populations and/or conducted in different geographic regions may lead to different results of the interrelationship between FSHR polymorphisms and PCOS.

Obesity and abdominal adiposity are typical clinical manifestations of PCOS patients. It has been reported that approximately 50 % of PCOS women are overweight or obese and most of them have the abdominal phenotype [22]. Based on BMI and WHR, the criteria used for obesities [23, 24], we investigated whether there was a correlation between obesity and the FSHR polymorphisms in PCOS patients. We have observed no correlation between the different obesity standard of PCOS patients and the genotypes of FSHR polymorphism. A panel of endocrine hormones was analyzed in relation to the genotypes of Ala307Thr and Ser680Asn polymorphisms in the PCOS patients. We have observed that the both FSHR polymorphisms were related to the level of FSH. In addition, the result showed that the Ser680Asn polymorphism was correlated with the level of PRL. Interestingly, there was no significant difference between the clinical pregnancy rate and the different genotypes of FSHR polymorphisms.

In conclusion, our results indicate that there is no association between the Ala307Thr and Ser680Asn polymorphisms of FSHR and PCOS patients in Han ethnical population from Shanxi Province in north China. In addition, the FSHR polymorphisms are associated with the levels of FSH and PRL.
